# Current Trends in Knee Arthroplasty: Are Italian Surgeons Doing What Is Expected?

**DOI:** 10.3390/medicina58091164

**Published:** 2022-08-26

**Authors:** Lorenzo Moretti, Michele Coviello, Federica Rosso, Giuseppe Calafiore, Edoardo Monaco, Massimo Berruto, Giuseppe Solarino

**Affiliations:** 1 Orthopaedic and Trauma Unit, Department of Basic Medical Sciences, Neurscience and Sense Organs, School of Medicine, AOU Consorziale Policlinico, University of Bari “Aldo Moro”, Piazza Giulio Cesare 11, 70124 Bari, Italy; 2 Ordine Mauriziano, Orthopaedics and Traumatology Department, Largo Turati 62, 10128 Turin, Italy; 3 Department of Orthopaedic and Trauma Surgery, Città di Parma Clinic, Piazzale Athos Maestri 5, 43123 Parma, Italy; 4 Orthopedic Unit, Kirk Kilgour Sports Injury Centre, S. Andrea Hospital, University of Rome Sapienza, 00189 Rome, Italy; 5 Chirurgia Articolare del Ginocchio, ASST Ospedale Gaetano Pini CTO, 20122 Milano, Italy

**Keywords:** orthopedic surgeon, planning, survey, total knee arthroplasty, unicompartmental knee arthroplasty

## Abstract

Objectives: The purpose of this study is to evaluate Italian surgeons’ behavior during knee arthroplasty. Materials and Methods: All orthopedic surgeons who specialized in knee replacement surgeries and were members of the Italian Society of Knee, Arthroscopy, Sport, Cartilage and Orthopedic Technologies (SIGASCOT) between January 2019 and August 2019 were asked to complete a survey on the management of knee arthroplasty. Data were collected, analyzed, and presented as frequencies and percentages. Results: One-hundred and seventy-seven surgeons completed the survey and were included in the study. Ninety-five (53.7%) surgeons were under 40 years of age. Eighty-five surgeons (48%) worked in public hospitals and 112 (63.3%) were considered “high volume surgeons”, with more than 100 knee implants per year. Postero-stabilized total knee arthroplasty was the most commonly used, implanted with a fully cemented technique by 162 (91.5%) surgeons. Unicompartmental knee arthroplasty (UKA) was a rarer procedure compared to TKA, with 77% of surgeons performing less than 30% of UKAs. Most common TKA pre-operative radiological planning included complete antero-posterior (AP) weight-bearing lower limb radiographs, lateral view and patellofemoral view (used by 91%, 98.9% and 70.6% of surgeons, respectively). Pre-operative UKA radiological images included Rosenberg or Schuss views, patellofemoral view and magnetic resonance imaging (66.1%, 71.8% and 46.3% of surgeons, respectively). One hundred and thirty-two surgeons (74.6%) included an AP weight-bearing lower limb X-ray one year after surgery in the post-operative radiological follow-up. Furthermore, 119 surgeons (67.2%) did not perform a post-operative patellofemoral view because it was not considered useful for radiological follow-up. There was no uniformity in the timing and features of post-operative follow-up, with 13 different combinations. Conclusions: Italian surgeons perform TKA more commonly than UKA. Pre-operative TKA planning is quite uniform rather than UKA planning. Despite literature evidence, there is no agreement on follow-up. It may be useful to create a uniform checklist, including correct timing and exams needed. This analysis is also part of a society surgical educational project for training doctor.

## 1. Introduction

Total knee arthroplasty (TKA) is a very common procedure in orthopedic surgery. It has increased by 162% over the past twenty years with approximately 250,000 primary and revision arthroplasties performed each year [1]. Main indications for knee arthroplasty remain primary or secondary osteoarthritis, rheumatoid arthritis in association or not with limitation in range of movement (ROM) or deformities. Modifiable and nonmodifiable prognostic factors were associated with the rate of unsatisfied patients from 5 to 40% after TKA [2,3]. Nowadays there are several TKA designs, which can be chosen depending on the patients’ age, expected activity level, pre-operative deformity and stability of the knee [4]. Particularly, different types of constraint can be used in primary TKA, from cruciate-retaining (CR) to postero-stabilized (PS) implants. There are also “high constrained” implants, such as condylar constrained, but they are normally reserved to revision TKA due to lower survivorship in primary TKA [5,6]. The first TKA designs did not include patellar implants, and they were characterized by high rate of post-operative anterior knee pain [7]. In the 1980s, patella-related complications accounted for up to 50% of complications following TKA [8]. Consequently, patellar resurfacing was introduced, but different complications were described. For these reasons, despite the number of studies, there is still some disagreement between surgeons who prefer patellar resurfacing, surgeons who never resurface the patella and surgeons who resurface it in selected cases [8,9,10,11,12]. In the case of degenerative arthritis involving only the medial or lateral compartment, unicompartmental knee arthroplasty (UKA) can be performed [13]. UKA needed the patient to have an intact anterior cruciate ligament and correctable knee alignment [14]. Similarly, isolated patellofemoral arthroplasty (PFA) can also be performed in selected cases with isolated patellofemoral osteoarthritis (PFOA) [15].

Both in TKA or UKA, correct pre-operative planning is mandatory to achieve a good outcome [16]. Different X-ray protocols have been described, but the most used are still the long-leg anteroposterior (AP) view for evaluation of the anatomical and mechanical axis [17,18,19,20], weight-bearing AP and lateral view of the knee and patellar view [21,22]. Magnetic resonance imaging (MRI) is usually not useful in TKA pre-operative planning, but it may be reasonable in UKA planning to evaluate cruciate ligaments status as well as cartilage status of the not-replaced compartment. Furthermore, it may also be useful in some revision surgery cases [23]. Careful pre-operative planning allowed the surgeon to predict possible difficulties or complications, need for higher constraint or specific instrumentation and it is mandatory to plan the surgeries [24]. Despite different studies on the correct pre-operative planning needed for both TKA and UKA, in practice there is still lack of uniformity. Current literature was investigated to compare our results with other large working groups, such as national registries, demonstrating similarities and differences. With regard to the Nordic Arthroplasty Register Association (NARA), that confirmed some Italian data, TKA was used as a primary implant (92%) in patients less than 65 y.o. (64%), while UKA and PFA were used less (3% and 1%, respectively) [25]. The experienced surgeons’ behaviors together with new learning technologies, such as augmented reality or cadaver labs, represent the focus of young surgeons trained by orthopedic societies [26,27].

The purpose of this study is to evaluate how Italian surgeons specialized in knee replacement behave in pre-operative planning and surgery. The authors, as active members of Italian orthopedic society and as active knee surgeons, guided this analysis as a starting point for educational pathways for young surgeons.

## 2. Materials and Methods

All orthopedic surgeons who specialized in knee replacement surgeries and were members of the Italian Society of Knee, Arthroscopy, Sport, Cartilage and Orthopedic Technologies (SIGASCOT) were asked to complete a survey on the current management of TKA and UKA, between January 2019 and August 2019, including pre-operative planning, implants used and characteristics of follow-up. Surgeons interviewed belong to the “knee surgery” specialized group of the society with 415 members. This is represented by doctors who spend more than half of their surgery in knee procedures with both arthroplasty and arthroscopy and who have followed high specialization courses.

**Scheme 1:**
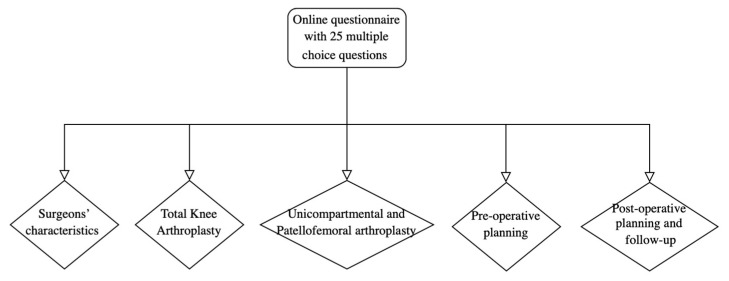
Flow diagram of questionnaire.

An online questionnaire was built using SurveyMonkey (Portland, OR, USA^®^), a free, open-source online survey tool. The 25 multiple choice questions were divided by subject into 5 parts: surgeons’ characteristics (i.e., age, volume of surgeries), TKA data (i.e., type of insert, cementation technique), UKA data (numbers of surgeries, type of implants), data regarding the pre-operative planning and follow-up (Scheme 1). The survey required approximately 10–12 min to be completed. Results from the survey were collected electronically and anonymously using SurveyMonkey (Portland, OR, USA^®^). All data were analyzed using SPSS 25.0 (IBM, Armonk, NY, USA). Data were evaluated using descriptive analysis, and they were presented as frequencies and percentages. The data used to support the findings of this study are available from the corresponding author upon request.

## 3. Results

**Figure 1:**
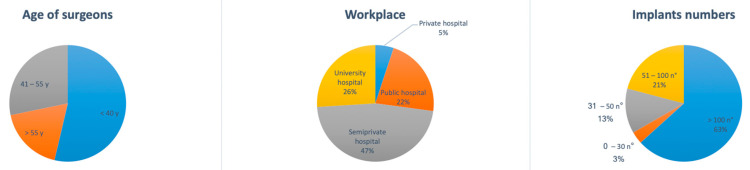
Demographic data, workplace and number of total annual implants. (y = year, n° = number).

One hundred and seventy-seven orthopedic surgeons (42.65%) completed the survey. Table 1 reports the survey in detail. Of the respondents, 46.9% worked in semiprivate hospitals, and 53.7% were under the age of 40. The demographic data, workplace and number of total annual implants included in the study are summarized in Table 2 and Figure 1.

With regard to total knee arthroplasty, most of the surgeons preferred a postero-stabilized (PS) (78%) cemented (91.5%) implant. Focusing on the type of cement preferred, most of the surgeons (45.2%) used antibiotic-loaded cement only in selected patients, while 35% of the surgeons always used antibiotic-loaded cement. The cruciate retaining (CR) insert was used by 20.3% of surgeons, while only 1.7% preferred the medial pivot insert. Nearly half of the surgeons preferred selected patellar resurfacing (46.3%). Data related to the TKA surgical technique are presented in Table 3.

**Figure 2:**
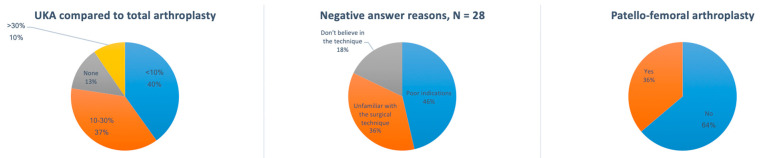
UKA and PFA related data. (N = number of answers).

With regard to unicompartmental knee arthroplasty (UKA) and patellofemoral arthroplasty (PFA), UKA was chosen less compared to TKA, with 40% of surgeons performing it in less than 10% of the cases, while 37.3% of surgeons performed UKA in 30% of the cases. Surgeons did not recognize a correct indication towards UKA (46.4%) and they were unfamiliar with the surgical technique (35.7%). During their activity, surgeons performed patellofemoral arthroplasty (PFA) in 36.2% of cases. UKA and PFA related data are summarized in order of frequency in Figure 2. 

With regard to radiological planning, characteristics of pre-operative planning were shared by most of the surgeons. For example, patellar view X-ray was required both in the total (70.6%) and in the unicompartmental knee arthroplasty (71.8%). Other routinely used radiographs were lateral view in 98.9% of cases and complete lower limb AP weight-bearing in 91% of cases. Some surgeons only used the latter for unicompartmental (1.7%) and others found it not useful (7.3%) due to its frequent execution errors (27.3%) or due to organization budget reasons (36.3%). However, in most of the cases the long-leg view was performed in bipodalic position (80.8%). Varus/valgus stress view was required only in 0.6% of cases, while Rosenberg or Schuss views in unicompartmental knee arthroplasty were requested in two thirds of cases (66.1%). The majority of surgeons recognized the importance of adequate pre-operative planning (80.2%). Regarding pre-operative MRI, 53.7% of surgeons did not require it and 37.3% required it only for UKA. Radiographic pre-operative data are summarized in order of frequency in Table 4.

With regard to post-operative follow-up, 74.6% of surgeons requested complete lower limb AP weight-bearing X-rays one year after knee replacement surgery. Only 23.2% did not use this view, mainly because they did not consider it useful (53,7%). Patellar view X-ray, very important in the pre-operative planning, was not required in the postoperative evaluation by 67.2% of the surgeons. When it was required, in one third of cases (32.8%), different views were performed, with the most commonly requested being the Merchant view at 45° of knee flexion (71% of the cases). The last two questions of the survey evaluated the post-operative follow-up. Unfortunately, there was no uniformity in the management of post-operative follow-up, with thirteen different combinations of timing and exams required, with none exceeding 35%. Radiographic and follow-up post-operative data are summarized in order of frequency in Table 5.

## 4. Discussion

The present survey was carried out in collaboration with the Italian Society of Knee, Arthroscopy, Sport, Cartilage and Orthopedic Technologies (SIGASCOT) and members of the Italian Society of Orthopedics (SIOT). The purpose of this study was to summarize Italian surgeons’ preferences in pre-operative planning, surgical technique and post-operative follow-up for TKA and UKA. The analysis of our data is the research subject of the Italian orthopedics society for educational pathway promotion for training doctors. The study was strongly supported by the society because it represents one of the starting points of training: the role of possible tutors and training centers. Every year the universities try to ensure proper education for their training doctors. The role of the societies in this educational program is to improve the choice of non-university training centers. The debate is still open regarding hospital type, the role of the tutor and the main features of the training center. An Italian survey example carried out by the same society with an education objective was published in 2017 [27].

Survey analysis revealed a population of young surgeons who worked mainly in semi-private hospitals and with “high volume” surgeries (more than 100 arthroplasties per year) (Table 2). Italian orthopedic training was made up of various resources, such as cad-lab, face-to-face and multimedia courses, and indexed journals [27]. This training leads young surgeons to being open to innovations and continuous updating.

TKA is still one of the most common orthopedic procedures, with good reported outcomes. However, almost 20% of the patients are still unsatisfied post-operatively and there is also a considerable complication rate, including periprosthetic joint infection [28,29]. Historically, UKA and PFA were considered “at risk” procedures, with high failure rates, mainly due to poor implants and surgical techniques [15,30]. However, recently, better outcomes were reported with new implants, with lower failure rate and higher patient satisfaction compared to TKA, especially for “high volume” surgeons [31]. However, despite improvement in UKA outcomes, Italian knee surgeons still performed more TKAs compared to UKAs or PFAs. The TKA planning was uniform among the surgeons, with a prevalence of the PS-TKA over the other types of implants. Instead, the planning of the UKA has proved uneven, with a major impact of surgeon volume on planning type. Finally, the post-operative follow-up was too different in terms of timing and type of radiological examination required. Emerged data respected the current literature review with lack of standardization of UKA. Many authors have recognized the importance of standing AP, lateral, Merchant and Rosenberg, stress views and MRI for UKA focusing attention on some radiographic prognostic values, but no recommendations have yet emerged from these results [31]. Furthermore, recent literature has also shown great interest in MRI in total knee arthroplasty. Some authors demonstrated that measuring the distances of Achilles tendon from the mechanical axis of lower limb in magnetic resonance imaging of the ankle helps towards correct alignment of the components in the coronal plane. The pre-operative planning role is fundamental for the success of the arthroplasty. Correct pre-operative studies, such as X-ray or MRI, are necessary for the evaluation of the axes of the knee [32]. Additionally, a new MRI-based approach for the analysis of thigh muscles was described to improve a patient-specific rehabilitation program [33]. Computerized tomography seems to be of great importance in the use of robot surgery, as demonstrated in a 2020 ESSKA review [34]. The robotic-assisted procedure had significantly lower postoperative pain score, significantly reduced time until hospital discharge and significantly better functional scores when compared with traditional surgery [34].

Similar society analyses have been conducted in the literature. Friederich et al. [35] investigated the computer-assisted use for total knee surgery among members of the European Society of Sports Traumatology Knee Surgery and Arthroscopy (ESSKA) and the Swiss Orthopedic Society (SGO-SSO). Authors described half of surgeons using this technology and the improvement in alignment of prosthesis was the most strongly cited reason for using a navigation system. Jaap et al. [36] studied realistic expectations for recovery one year after TKA among Dutch orthopedic surgeons using a hospital for special surgery score. They concluded that greatest improvement was predicted for the items “pain relief” and “walking short distances”. The British Association for Surgery of the Knee in conjunction with the James Lind Alliance investigated assessment, management and rehabilitation of patients with persistent symptoms after knee arthroplasty including patients, surgeons, anesthetists, nurses, physiotherapists and researchers. They concluded that the top ten research priorities focused on pain, infection, stiffness, health service configuration, surgical and non-surgical management strategies and outcome measures [37]. Alexander et al. [38], instead, surveyed orthopedic surgeons affiliated with the American Association of Hip and Knee Surgeons to inquire into the global impact of the COVID-19 pandemic on patient care. They described that all respondents noted their practices had been reduced and 70% of the surgeons canceled elective procedures. Our study, unlike the previous ones, examined Italian knee surgeons’ behaviors. The questionnaire is completed with a pre- and post-operative analysis, including the main types of implants available. Furthermore, it is important to underline the careful selection of the interviewees, as members of a specialized group of society.

Considering the type of implants used, data from this study can be compared to different international arthroplasty registers. The Nordic Arthroplasty Register Association (NARA) confirmed some Italian data. TKA was used as a primary implant (92%) in patients younger than 65 y.o. (64%), while UKA and PFA were less used (3% and 1%, respectively). From our data, UKA was chosen in less than 10% of cases and PFA was implanted in 36.2%. NARA demonstrated that cemented fixation was used in 92% of all TKAs as in our survey (91.5%). The patella was resurfaced in 22% of cases, while our survey showed almost double the value. Hybrid fixation was used in 5% of all TKAs [25] as with our data (2.8%). Similar data emerged from the Italian register of arthroplasty (RIAP). TKA was chosen more often than UKA (83.6% and 16.4%, respectively), as emerged from our data. Cemented fixation was used in 66.9% of TKAs and 65.7% of all UKAs, slightly lower values than ours. Hybrid fixation was used in 3.4% and 6.9% of TKAs and UKAs, respectively, as with our data. Patellar resurfacing was only used in 12.1% of TKAs and 1.8% of UKAs [39]. These data seem lower than ours due to the “Selected patients” answers to question 10. This created a bias regarding the absolute value of resurfacing, masking data similar to the Italian register

In the United States in 2016, 50% of surgeons preferred postero stabilized (PS) implants, and cruciate retaining (CR) was the second design commonly used, with almost 42% of total procedures [40]. These data are in line with this study, in which PS implants are preferred by most of the surgeons. This is probably due to contraindication to CR implants, such as posterior cruciate ligament (PCL) insufficiency, significant coronal deformity, extensor mechanism deficiency, posterolateral instability and inflammatory arthritis. Moreover, PS-TKA is generally easier to perform in most surgical situations without concern for obtaining appropriate tension on the PCL [41].

Cemented TKA guarantees good clinical outcomes with a long-term survival rate of up to 99% in comparison to a survival rate of up to 97% documented in cementless TKA [6,42].

Initial total knee arthroplasty designs did not include patellar implants, in fact high rate of anterior knee pain was found following these operations [7]. Modern TKA designs have all-polyethylene patellar implants, as the older metal-backed implants had high rates of wear and loosening [43]. Despite this innovation there are still complications related to the patellar implant, so much so that patellar resurfacing has been subject to controversy for several years [44].

Different implant types were studied by researchers but not included in our survey due to their non-popularity. Sabatini et al. [45] reviewed the second generation of bicruciate-retaining TKA. They summarized that in cases of high demand, end-stage bi- or tricompartmental knee arthritis, coronal malalignment <15°, ACL integrity and minimal ROM reduction (< 5/10°), this procedure could be a valid alternative to TKA or UKA [45].

Custom TKA, as new implants, are useful in cases of anatomical and functional variability. They added asymmetry and sizes to the existing implants. Actually, our understanding of the relation between the dynamic aspects of gait and position and form of the knee implants is lacking. Custom arthroplasties could also address these conceptual and practical difficulties due to robotics and artificial intelligence [46].

New materials for use are currently being researched. Ultra-high molecular weight polyethylene (UHMWPE) is widely chosen for its biomechanical characteristics. For reducing the polyethylene wear, one of the efforts is to investigate the selection of metal materials. Jamari et al. [47] analyzed the relationship between the polyethylene and metals via finite element analysis. They described, in total hip arthroplasty, that titanium alloy is able to reduce cumulative contact pressure if compared to stainless steel and cobalt chromium molybdenum on UHMWPE [47].

As emerged from the survey, there is no uniformity in the management of post-operative follow-up, with thirteen different answers. Different studies confirmed the importance of a complete post-operative follow-up, but also in the literature there is no uniformity in timing of evaluation, with variability ranging from 3 to 6 months [15].

The novelty of this study is the careful analysis of the Italian surgical situation compared to other countries. With the high number of annual procedures, the state could play a key role in research and innovation.

This study had some limitations. The number of participants in the study was low compared to those registered with the society. The sample was not homogeneous in terms of age, location of work and number of prostheses, leading to some bias. The analysis does not consider the new technologies present in the literature such as robots, patient-specific instrument or fluoroscopy-guided surgery. On the other hand, thanks to a complete questionnaire, the study analyzes the behavior of Italian surgeons and provides society with the starting point for the educational analysis of young surgeons. Although the questionnaire was quite complete, further aspects could be investigated about the surgical technique, such as surgical time, surgical approach and others.

An analysis is currently underway with similar questions addressed to training doctors, with the aim of comparing the answers of this analysis. This second survey represents the end of the research project which will be followed by the conception of educational practical courses, together with other projects already in progress.

## 5. Conclusions

Despite the improvement in UKA and PFA, we conclude that TKA still remains the preferred surgical option for Italian surgeons. PS cemented implants are the most commonly used, and patellar resurfacing was selected by most of the surgeons. Pre-operative planning is consistent with those reported in the literature and there is some agreement between surgeons, especially for TKA planning. When evaluating data regarding UKA, there is less agreement in pre-operative X-ray evaluation, with some surgeons requesting MRI and some surgeons preferring stress X-rays. Similar to the literature, there is absolutely no agreement in post-operative follow-up, both in terms of timing or radiological evaluation for both TKA and UKA. Considering these data, it may also be useful to promote some educational programs for training doctors after knowing their starting point.

## Figures and Tables

**Table 1:** Questions asked and possible responses.

	Questions	Possible Responses
1.	How old are you?	<40 y
		41–55 y
		>55 y
2.	Where do you work, as your main activity?	Public hospital
		Semiprivate hospital
		Private hospital
		University hospital
3.	How many total knee arthroplasties are performed each year in the hospital you work at?	0–30 n°
		31–50 n°
		51–100 n°
		>100 n°
4.	What kind of first implant do you mainly perform?	PS
		CR
		Medial Pivot
5.	What kind of fixation do you perform?	Cemented arthroplasty
		Uncemented arthroplasty
		Hybrid arthroplasty
		Cemented and uncemented
		Cemented or uncemented
6.	If you answered cemented to the previous question: what type of cement?	Antibiotic cement always
		Antibiotic in revision surgery only
		Antibiotic in selected patient only
		Antibiotic cement never
7.	Do you perform unicompartmental knee arthroplasty? If so, compared to total arthroplasty?	None
		>10%
		10–30%
		>30%
8.	If you answered NO to the previous question, explain why:	Open answer
9.	Do you perform femoropatellar arthroplasty?	Yes
		No
10.	Do you perform a patella arthroplasty?	Hardly ever
		Almost always
		Selected patient
		90% in woman
11.	Do you perform patellar view X-ray in the preoperative study of total knee arthroplasty?	Yes
		No routinely
12.	Do you perform patellar view X-ray in the preoperative study of unicompartmental knee arthroplasty?	Yes
		No routinely
13.	Do you perform lateral view in the preoperative study?	Yes
		No routinely
14.	Do you perform AP weight-bearing of the whole lower limb view in the preoperative study?	Yes
		No routinely
		UKA only
15.	If you answered NO to the previous question, explain why:	Useless
		Organizational budget reasons
		Severe axis changes
		Major deformities
		Execution errors are frequent
16.	If you answered YES to the previous question, how do you request it?	Bipodalic position
		Monopodalic position
		Monopodalic for UKA
17.	Do you perform varus/valgus stress view?	Yes
		No
18.	Do you perform Rosenberg or Schuss views in the unicompartmental knee arthroplasty preoperative study?	Yes
		No
19.	Do you regularly perform preoperative planning?	Yes
		No
20.	Do you regularly perform MRI in the preoperative study?	Yes
		No
		UKA only
21.	One year after knee replacement surgery, do you require AP weight-bearing of the whole lower limb view?	Yes
		No
		Selected patient
		Pain UKA only
		For research only
		Severe axis changes
22.	If you answered NO to the previous question, explain why:	Useless
		Organizational budget reasons
		Useless if the patient has no pain
		Execution errors are frequent
23.	Do you regularly perform postoperative patellar view?	Yes
		No
24.	If so, which one?	Merchant (45° view)
		Ficat (30-60-90° view)
		Baldini (under bearing view)
		30° view
25.	When do you perform postoperative radiographic follow-up (you can choose multiple answers)?	1 m
		3 m
		6 m
		12 m
26.	Comments and advice	Open answer

(y = year, n° = number, PS = postero stabilized, CR = cruciate retaining, m = month).

**Table 2:** Demographic data, workplace and number of total annual implants.

How Many Total Knee Arthroplasties Are Performed Each Year in the Hospital You Work At?	<40 y	>55 y	41–55 y	Total
**Semiprivate hospital**	**22.0%**	**11.3%**	**13.6%**	**46.9%**
0–30 n°	1.1%	0.6%	0.0%	1.7%
31–50 n°	1.1%	1.7%	0.0%	2.8%
51–100 n°	2.8%	1.1%	4.0%	7.9%
>100 n°	17.0%	7.9%	9.6%	34.5%
**University hospital**	**19.8%**	**1.7%**	**4.5%**	**26.0%**
31–50 n°	4.0%	0.0%	1.1%	5.1%
51–100 n°	3.4%	0.0%	1.1%	4.5%
>100 n°	12.4%	1.7%	2.3%	16.4%
**Public hospital**	**9.6%**	**4.0%**	**8.5%**	**22.0%**
0–30 n°	0.6%	0.0%	0.6%	1.1%
31–50 n°	1.1%	0.6%	2.3%	4.0%
51–100 n°	4.0%	1.7%	2.8%	8.5%
>100 n°	4.0%	1.7%	2.8%	8.5%
**Private hospital**	**2.3%**	**1.1%**	**1.7%**	**5.1%**
0–30 n°	0.6%	0.0%	0.0%	0.6%
31–50 n°	0.0%	0.6%	0.0%	0.6%
>100 n°	1.7%	0.6%	1.7%	4.0%
**Total**	**53.7%**	**18.1%**	**28.2%**	**100.0%**

(y = year, n° = number).

**Table 3:** Total knee arthroplasty related data.

	Frequency (%)
**Type of first implant**	
Postero-stabilized	138 (78%)
Cruciate retaining	36 (20.3)
Medial pivot	3 (1.7)
**Type of fixation**	
Cemented arthroplasty	162 (91.5)
Uncemented arthroplasty	8 (4.5)
Hybrid arthroplasty	5 (2.8)
Cemented and uncemented	1 (0.6%)
Cemented or uncemented	1 (0.6%)
**Type of cement**	
Antibiotic in selected patient only	80 (47.6)
Antibiotic cement always	62 (36.9%)
Antibiotic cement never	23 (13.7%)
Antibiotic in revision surgery only	3 (1.8%)
**Patellar resurfacing**	
Selected patients	82 (46.3%)
Almost always	49 (27.7)
Hardly ever	45 (25.4%)
90% in woman	1 (0.6%)

**Table 4:** Radiographic pre-operative data.

	Frequency (%)
**Patellar view X-ray in the pre-operative of TKA**	
Yes	125 (70.6%)
No routinely	52 (29.4%)
**Patellar view X-ray in the pre-operative of UKA**	
Yes	127 (71.8%)
No routinely	50 (28.2%)
**Lateral view in the pre-operative**	
Yes	175 (98.9%)
No routinely	2 (1.1%)
**AP weight-bearing of the whole lower limb view in the pre-operative**	
Yes	161 (91%)
No routinely	13 (7.3%)
UKA only	3 (1.7%)
**Negative answer reasons**	N = 11
Organizational budget reasons	4 (36.3%)
Execution errors are frequent	3 (27.3%)
Useless	2 (18.2%)
Severe axis changes	1 (9.1%)
Major deformities	1 (9.1%)
**How do you request it?**	N = 167
Bipodalic position	143 (85.6%)
Monopodalic position	16 (9.6%)
Monopodalic for UKA	8 (4.8%)
**Varus/valgus stress view**	
No	176 (99.4%)
Yes	1 (0.6%)
**Rosenberg or Schuss views in the UKA pre-operative**	
Yes	117 (66.1%)
No	60 (33.9%)
**Pre-operative planning**	N = 177
Yes	142 (80.2%)
No	35 (19.8%)
**MRI in the pre-operative**	
No	95 (53.7%)
UKA only	66 (37.3%)
Yes	16 (9%)

(TKA = total knee arthroplasty, UKA = unicompartmental knee arthroplasty, AP = antero-posterior, MRI = magnetic resonance imaging, N = number of answers).

**Table 5:** Radiographic and follow-up post-operative data.

	Frequency (%)
**AP weight-bearing of the whole lower limb view one year after surgery**	
Yes	132 (74.6%)
No	41 (23.2%)
Selected patient	1 (0.6%)
Pain UKA only	1 (0.6%)
For research only	1 (0.6%)
Severe axis changes	1 (0.6%)
**Negative answer reasons**	N = 41
Useless	22 (53.7%)
Organizational budget reasons	13 (31.7%)
Execution errors are frequent	5 (12.2%)
Useless if the patient has no pain	1 (2.4%)
**Post-operative patellar view**	
No	119 (67.2%)
Yes	58 (32.8%)
**Kind of post-operative patellar view**	N = 62
Merchant (45° view)	44 (71%)
Baldini (under bearing view)	9 (14.5)
Ficat (30–60–90° view)	8 (12.9%)
30° view	1 (1.6%)
**Post-operative radiographic follow-up (months)**	N = 177
1–3–6–12 m	56 (31.6%)
1–6–12 m	29 (16.4%)
3–6–12 m	27 (15.3%)
3–12 m	19 (10.7%)
1–12 m	13 (7.3%)
1 m	12 (6.8%)
3 m	8 (4.5%)
6–12 m	6 (3.4%)
1–6 m	3 (1.7%)
12 m	2 (1.1%)
1–3–6 m	1 (0.6%)
6 m	1 (0.6%)
**Comments and advice**	N = 2
First check-up 45 days	1 (50%)
Long plate X-ray complete AP weight-bearing radiograph	1 (50%)

(AP = antero-posterior, UKA = unicompartmental knee arthroplasty, N = number of answers).

## Data Availability

The data presented in this study are available on request from the corresponding author.
